# Supplementary material for: PRL/microRNA-183/IRS1 Pathway Regulates Milk Fat Metabolism in Cow Mammary Epithelial Cells
Source: Genes (Basel). 2020 Feb 13;11(2):196. doi: 10.3390/genes11020196 (PMC7073568; doi:10.3390/genes11020196)
Supplement: Supplementary file 1 [file genes-11-00196-s001.pdf]

Table S1: Bta-mir-183 sequence

miRNA primer name	Primer sequences
>bta-miR-183 MIMAT0003813	UAUGGCACUGGUAGAAUUCACUG

Table S2: Primer sequence of gene

*Primer sequences(5'--3')	*The primer name
F: GGAGCTTATCCAGGCCAATG	F:ATGL
R: TGCGGGCAGATGTCACTCT	R:ATGL
F: GTACAGATGCAGCCTCATTTCC	F: CD36
R: TGGACCTGCAAATATCAGAGGA	R:CD36
F: CCAGCTGACAGCTCCATTGA	F: SREBP1
R: TGCGCGCCACAAGGA	R:SREBP1
F: AGGACACTTGCCACCTCATTC	F: ABCG1
R: TTGGAGTCTGGTTCCCTCTTGTA	R: ABCG1
F: CCATCGCCTGTGGAGTCAC	F: SCD
R: GTCGGATAAATCTAGCGTAGCA	R:SCD
F: AAGGACCTCTACGCCAACACG	F: CPT1
R: TTTGCGGTGGACGATGGAG	R:CPT1
F: CAGCTGGCCAAATACCTTCAA	F:UXT
R: CAGCTGGCCAAATACCTTCAA	R:UXT
F: CTCCAACCTCAACCACTACGG	F: ABCA1
R: GGGGAATCACAGAAGCAGCC	R:ABCA1
F: GGGCTCCACCACCGTGTTCCA	F:FASN
R:GCTCTGCTGGGCCTGCAGCTG	R:FASN
F: GGGAGCACTACAAACGCAACG	F:HSL
R:TGAATGATCCGCTCAAACTCG	R:HSL
F: TCAGTCGGGAAGCGAATGGA	F:DNMT1
R:CTGGAGACAGGTTTGGGGGG	R:IDNMT1

Table S3: IRS1 siRNA sequence

	sense（5'-3'）	antisense（5'-3'）
IRS1-siRNA	GGAAAUAUAUCCUGGACAATT	UUGUCCAGGAUAUAUUUCCTT

Table S4: Primers for dual luciferase reporter gene

*Primer sequences(5'--3')	*The primer name
ccgctcgagTTGCGTTGGGTGGAGAGAGT	IRS1-up
ATTGGCAATTGAATGGAAGCAcgccggcgtataaa	IRS1-Down
GTAATATAATAAGAATCATATTTTTTTGATTTTGGTTCTCAATTG	IRS1-overlap
